# Venetoclax with azacitidine targets refractory MDS but spares healthy hematopoiesis at tailored dose

**DOI:** 10.1186/s40164-019-0133-1

**Published:** 2019-04-16

**Authors:** Stefanie Jilg, Richard T. Hauch, Johanna Kauschinger, Lars Buschhorn, Timo O. Odinius, Veronika Dill, Catharina Müller-Thomas, Tobias Herold, Peter M. Prodinger, Burkhard Schmidt, Dirk Hempel, Florian Bassermann, Christian Peschel, Katharina S. Götze, Ulrike Höckendorf, Torsten Haferlach, Philipp J. Jost

**Affiliations:** 1 Medical Department III for Hematology and Oncology, Klinikum rechts der Isar, Technische Universität München, Ismaninger Strasse 22, 81675 Munich, Germany; 2 Department of Internal Medicine 3, University Hospital Grosshadern, Ludwig-Maximilians-Universität (LMU), 81377 Munich, Germany; 3 Department of Orthopedic Surgery, Klinikum rechts der Isar, Technische Universität München, Munich, Germany; 4 Gemeinschaftspraxis Hämato-Onkologie Pasing, Munich, Germany; 5 Onkologisches Zentrum Donauwörth, Donauwörth, Germany; 6 German Consortium for Translational Cancer Research (DKTK) of the German Cancer Research Center (DKFZ), Heidelberg, Germany; 7 Munich Leukemia Laboratory (MLL), Munich, Germany

**Keywords:** MDS, Venetoclax, Combination therapy, Hematotoxicity, HMA failure

## Abstract

Patients with Myelodysplastic Syndromes (MDS) and secondary Acute Myeloid Leukemia (sAML) have a very poor prognosis after failure of hypomethylating agents (HMA). Stem cell transplantation is the only effective salvage therapy, for which only a limited number of patients are eligible due to age and comorbidity. Combination therapy of venetoclax and azacitidine (5-AZA) seems to be a promising approach in myeloid malignancies, but data from patients with HMA failure are lacking. Furthermore, a considerable concern of combination regimens in elderly AML and MDS patients is the toxicity on the remaining healthy hematopoiesis. Here, we report in vitro data showing the impact of venetoclax and 5-AZA, alone or in combination, in a larger cohort of MDS/sAML patients (n = 21), even after HMA failure (n = 13). We especially focused on the effects on healthy hematopoiesis and the impact on colony forming capacity as a parameter for long-term effects. To the best of our knowledge, we show for the first time that venetoclax in combination with capped dose of 5-AZA targets cell malignancies, while sparing healthy hematopoiesis.

**Electronic supplementary material:**

The online version of this article (10.1186/s40164-019-0133-1) contains supplementary material, which is available to authorized users.

Patients with Myelodysplastic Syndromes (MDS) and secondary Acute Myeloid Leukemia (sAML) have a very poor prognosis after failure of hypomethylating agents (HMA). For these patients, stem cell transplantation represents the only effective salvage therapy, for which only a limited number of patients are eligible due to age and comorbidity. Further durable treatment options are completely lacking at the moment [1].

A Phase 1b multicentre study (M15-522) has been initiated to determine the safety of venetoclax treatment, as monotherapy or in combination with 5-azacitidine (5-AZA), in subjects with relapsed/refractory MDS. This approach is based on in vitro findings showing high efficacy of venetoclax monotherapy in high-risk MDS/sAML [2, 3] and synergistic effects of venetoclax and 5-AZA in primary samples [4].

Venetoclax blocks the activity of the pro-survival BCL-2 protein, priming the cells for apoptosis. Flow cytometry analysis shows an increase in BCL-2 levels and a decrease in MCL-1 levels after HMA treatment, resulting in a profile even more favorable for treatment with venetoclax (data not shown). Therefore, venetoclax treatment may reduce the apoptotic threshold in MDS or AML cells, leading to an improved response to HMA, even in cells previously resistant to HMA treatment [4, 5]. Interestingly, recently published data show that combination therapy of venetoclax and 5-AZA azacitidine might even eradicate leukemia stem cells by disrupting the metabolic machinery [6].

In vivo data from a phase-1b study show promising response rates of 5-AZA or decitabine in combination with venetoclax in elderly AML patients [7, 8] and patients with related myeloid malignancies [9].

However, a considerable concern of combination regimens in elderly AML and MDS patients is the toxicity on the remaining healthy hematopoiesis. DiNardo et al. report toxicity as manageable, yet relatively high rates of febrile neutropenia were observed [7, 8], especially in patients with relapsed/refractory disease (72%) [9]. Furthermore, similar data were presented at ASH 2017 [10] and EHA 2018 [11] for combination therapy of venetoclax with low-dose chemotherapy.

The same safety concern was also detected in an ongoing clinical trial (Abbvie M15-531 trial), in which higher-risk MDS patients are being treated with a combination therapy of venetoclax and 5-AZA. Due to increased rate of toxic side-effects, the daily dose of venetoclax had to be reduced from 800 to 400 mg in all arms.

Of note, as we have learned from clinical reality, co-treatment with CYP3A4 inhibitors alters venetoclax plasma concentration, maybe aggravating cytotoxic side effects [12].

Here, we report in vitro data, showing the effects of the treatment with venetoclax and 5-AZA, alone or in combination, in a cohort of MDS/sAML patients (n = 21) with a mean age of 72.14 (range 57–84 years), including a subset of patients resistant to HMA treatment (n = 13, referred to as “HMA failure”), defined according to the 2006 IWG (International Working Group) response criteria (Additional file 1: Table S1) [mean age 70.08 (range 57–80 years)]. As healthy controls, we used bone marrow mononuclear cells (BMMNCs) isolated from the femoral bone of 19 elderly patients undergoing surgical hip replacement [mean age: 64.96 years (range: 49–85 years)]. To investigate the impact on healthy hematopoiesis, we evaluated BMMNCs viability after 72 h treatment (short-term) and colony forming capacity (long-term).

First, we analyzed the effects of venetoclax and 5-AZA treatment in healthy, age-matched bone marrow samples, to tailor our combination dosages towards a more tolerable regime. Primary BMMNCs were cultivated in growth-factor enriched media as previously described [2]. Viability after 72 h-treatment was determined by flow cytometry using Annexin V and 7-amino-actinomycin D (7-AAD). To analyze colony-forming capacity, cells were pre-treated in liquid culture for 72 h, then transferred into growth-factor enriched methylcellulose and evaluated after 10–14 days.

For all our in vitro experiments, we used venetoclax at 1 µM concentration, which corresponds to the clinically used standard dose of 400 mg [13]. In line with our previously published data [2, 3],

1 µM venetoclax had marginal cytotoxic effects on the bulk of BMMNCs from healthy elderly subjects (Fig. 1a). Increasing concentrations (1–10 µM) of 5-AZA showed a dose-dependent toxicity for healthy bulk BMMNCs (Fig. 1a). When analyzing combination therapy of 1 µM venetoclax with increasing concentrations of 5-AZA, we detected a significant decrease in cell viability (to 40.5%). These results suggest a synergistic toxic effect of the combination therapy on the hematopoietic compartment (Fig. 1a).

**Fig. 1:**
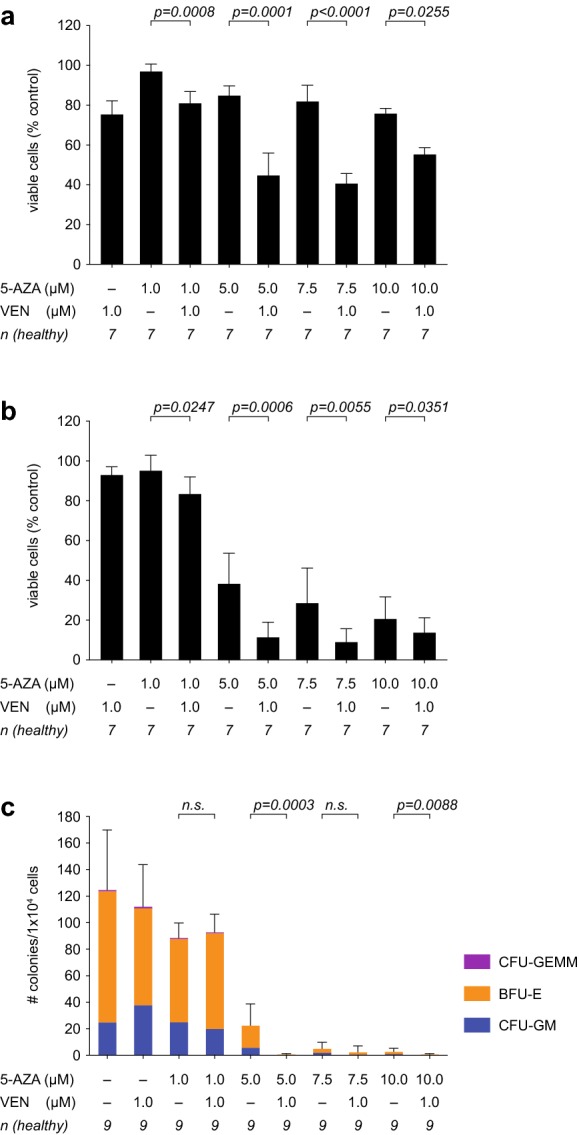
Healthy hematopoiesis is affected by higher dosage combination therapy. **a**, **b** Bone marrow mononuclear cells (BMMNCs) from healthy, elderly donors were treated for 72 h with the indicated concentrations of venetoclax (VEN) and 5-azacitidine (5-AZA), alone or in combination. The viability of bulk bone marrow cells (**a**) (n = 7) or purified CD34^+^ cells (**b**) (n = 7) was measured by flow cytometry using Annexin V and 7AAD staining. Data are presented as mean ± standard deviation (SD) of the ratio between viable cells after a 72 h treatment with drug or vehicle (DMSO). One-way ANOVA resulted in p < 0.0001 for total BMMNC (**a**) and p < 0.0001 for the CD34^+^ compartment. Results from post hoc pairwise comparison are reported in the figure. **c** BMMNCs (1 × 10^4^) from 9 individual healthy donors were plated in methylcellulose after 72 h of treatment with 1 µM venetoclax and 5-AZA (1 µM, 5 µM, 7.5 µM or 10 µM as indicated), alone or in combination. The total number of colonies, distinguishing between colony-forming units (CFU) of the multi-potential granulocytic–erythroid–macrophagic–megakaryocytic lineage (CFU-GEMM), the granulocytic–macrophagic lineage (CFU-GM), and the burst-forming units-erythroid lineage (BFU-E) were determined at day 14. Experiments were performed in duplicates. One-way ANOVA resulted in p < 0.0001. The results from post hoc pairwise comparison are reported in figure

To better delineate the toxic effects on hematopoiesis, we focused on the stem/progenitor compartment (CD34^+^) of healthy control subjects. We found that 5 µM, 7.5 µM and 10 µM 5-AZA decreased the viability of CD34^+^ cells by more than 50% (Fig. 1b). Cell toxicity was further increased by venetoclax, decreasing viability of the stem/progenitor compartment to below 20% (Fig. 1b).

These adverse effects were even more striking when analyzing the clonogenic potential of stem/progenitor cells in a colony-forming assay. Treatment with 5-AZA at 5 µM, 7.5 µM and 10 µM significantly decreased colony numbers in healthy controls. The addition of venetoclax to 5-AZA (5 µM or 10 µM) further decreased the colony forming capacity, resulting in an almost complete eradication of hematopoietic colonies (Fig. 1c). The combination therapy in vitro particularly affected the granulopoiesis, which is likely associated with the clinically-observed neutropenia (Fig. 1c). Taken together these data provide evidence of a substantial impact of venetoclax-5-AZA co-treatment on the healthy hematopoiesis. Therefore, the combination of venetoclax with 5-AZA may represent a feasible approach only when 5-AZA is capped at a dose of 1 µM in order to avoid toxic side-effects.

The suggested dose for 5-AZA is 75 mg/m^2^ of body surface, administered daily for 7 days per cycle. In daily clinical routine, 100 mg or 200 mg are the dosages applied. Therefore, patients with a body surface higher than 1.52 m^2^ receive 200 mg 5-AZA at least on 1 day of the treatment cycle. Pharmacokinetic data for doses of precisely 100 mg and 200 mg are not available in public databases. However, data are available for doses from 126 mg up to 165 mg (after subcutaneous administration) [13]. For these doses, the mean maximum concentration in the peripheral blood (C_max_) is 750 ± 403.3 ng/ml, ranging from 346.7 ng/ml (1.42 µM) to 1153.3 ng/ml (4.72 µM) [14].

In our in vitro setting, a combination treatment corresponding to a clinical regimen of 400 mg venetoclax plus 100 mg 5-AZA daily represents a low-toxic approach. The use of higher dosages of 5-AZA as part of a combination therapy might be critically discussed in order to avoid toxic side-effects on healthy hematopoiesis.

To better elucidate the clinical relevance of the combination therapy, we tested venetoclax and 5-AZA on primary malignant MDS and sAML cells. Surprisingly, when combined with venetoclax, low-dose 5-AZA (1 µM) was as effective as high-dose 5-AZA (10 µM) in reducing primary malignant MDS/sAML cells (Fig. 2a). Specifically, 13 out of 19 patients (68.4%) showed a reduction in viability below 65% (compared to soluble control) with high-dose 5-AZA, and 5 out of 8 patients (62.5%) using low-dose (Fig. 2a).

**Fig. 2:**
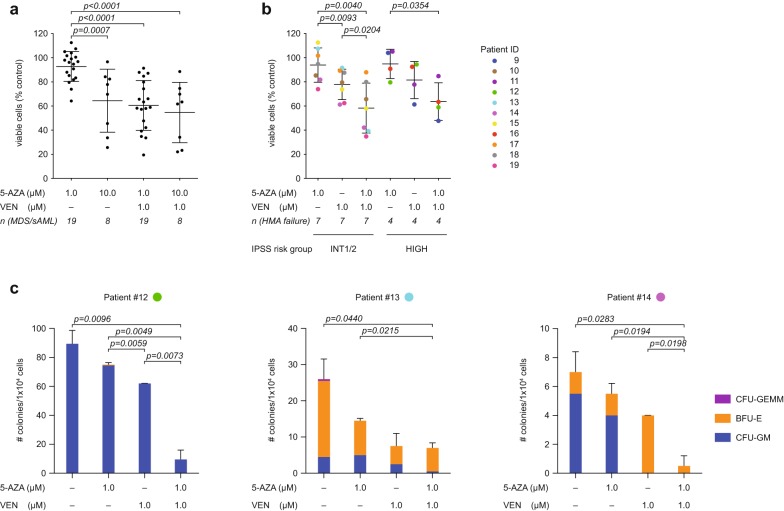
Combination of 5-AZA and venetoclax is highly effective after HMA failure despite dose adjustment. **a** CD34^+^ BMMNCs from patients with Myelodysplastic Syndromes (MDS) or secondary acute myeloid leukemia (sAML) were treated for 72 h with venetoclax (VEN), 5-azacitidine (5-AZA), alone or in combination. Cell viability was measured by flow cytometry using Annexin V and 7AAD staining. Data are presented as mean ± standard deviation (SD) of the ratio between viable cells after a 72 h treatment with drug or vehicle (DMSO). One-way ANOVA resulted in p < 0.0001. The results from post hoc pairwise comparison are reported in figure. **b** CD34^+^ BMMNCs from patients with MDS intermediate risk (INT) (according to IPSS) or sAML and failure of hypomethylating agent (HMA) were treated for 72 h with venetoclax (VEN), 5-AZA or the combination of both at the indicated concentrations. Cell viability was measured by flow cytometry using Annexin V and 7AAD staining. Data points representing the same patient are depicted in the same colour. Shown is the ratio between viable cells after a 72 h inhibitor or vehicle treatment (DMSO) with the mean and standard deviation (SD). One-way ANOVA resulted in p = 0.0025. The results from post hoc pairwise comparison are reported in figure. **c** BMMNCs (1 × 10^4^) from patient #12, #13 and #14 as described in **b** with sAML or high-risk MDS (according to IPSS) after HMA failure were plated in methylcellulose after 72 h of treatment with Venetoclax (VEN), 5-AZA or the combination of both at the indicated concentrations. The total number of colonies was determined at day 10 to 14. Experiments were performed in duplicates. One-way ANOVA resulted in p = 0.0005 for patient #12, p = 0.015 for patient #13, and p = 0.0068 for patient #14. The results from post hoc pairwise comparison are reported in figure

Patients with prior HMA failure are of special interest as treatment options for these patients remain dismal [1, 15]. Therefore, we analysed samples from the “HMA failure” group, treated with 1 µM venetoclax or 1 µM 5-AZA, alone or in combination. In 11 samples, cell numbers were sufficient enough to analyse all treatments in parallel. In a small number of patients (3 out 11, 27.3%), single-agent venetoclax showed beneficial effects (Fig. 2b). However, the combination of venetoclax with 5-AZA elicited a significant reduction in cell viability independent of IPSS grade and response to individual reagent (Fig. 2b).

To investigate the long-term effects of the combination therapy, we evaluated colony formation capacity in three individual “HMA failure” patients. In these patients, combination treatment of low-dose 5-AZA and venetoclax showed a profound effect. These results further support the notion that dose adjustments in the combination treatment will be beneficial, specifically for the high-risk group of “HMA failure” patients with MDS or sAML (Fig. 2c). To the best of our knowledge, complex karyotype or adverse mutational profile (listed in Additional file 1: Table S1) had no negative impact on treatment response (data not shown).

In summary, our data strengthen the scientific rationale of a therapeutic approach with a combination therapy using 5-AZA and venetoclax in patients with MDS/sAML, overall in “HMA-failure” patients. These patients have an extremely poor prognosis and new therapeutic strategies are urgently needed. Interestingly, in our in vitro setting even lower-dose 5-AZA shows a valuable cytotoxicity on the malignant cell when combined with venetoclax. Toxicity on the non-malignant progenitors was significantly reduced. However, further clinical trials will be needed to test the impact of our work on clinical reality. Here, a feasible approach might be capping the daily dosage of 5-AZA at 100 mg/day when used in a venetoclax combination regimen. This study would not only evaluate toxicity, but the effect of lower-dose 5-AZA on patients` outcome: less toxicity at the cost of efficacy may not be an acceptable trade off.

## Additional files

**Additional file 1: Table S1.** Clinical characteristics of MDS/sAML patients contributing samples. This table shows the clinical and molecular characteristics of MDS and sAML patients utilized for ex vivo treatment with venetoclax and 5-azacitidine in direct comparison.

**Additional file 2.** Patients and Methods.

## Abbreviations

BFU-E: burst-forming units-erythroid lineage
BMMNC: bone marrow mononuclear cells
CFU: colony-forming units
CFU-GM: colony-forming units of the the granulocytic-macrophagic lineage
CFU-GEMM: colony-forming units of the multi-potential granulocytic–erythroid–macrophagic–megakaryocytic lineage
HMA: hypomethylating agents
IWG: International Working Group
MDS: myelodysplastic syndromes
sAML: secondary acute myeloid leukemia
VEN: venetoclax
5-AZA: 5-azacitidine
7-AAD: 7-amino-actinomycin D
