# Supplementary material for: Venetoclax with azacitidine targets refractory MDS but spares healthy hematopoiesis at tailored dose
Source: Exp Hematol Oncol. 2019 Apr 16;8:9. doi: 10.1186/s40164-019-0133-1 (PMC6469098; doi:10.1186/s40164-019-0133-1)
Supplement: Supplementary file 1 — Additional file 1: Table S1. Clinical characteristics of MDS/sAML patients contributing samples. This table shows the clinical and molecular characteristics of MDS and sAML patients utilized for ex vivo treatment with venetoclax and 5-azacitidine in direct comparison. [file 40164_2019_133_MOESM1_ESM.pdf]

Additional File 1 Table S1: Clinical characteristics of MDS/sAML patients contributing samples. This table shows the clinical and molecular characteristics of MDS and sAML patients utilized for *ex vivo* treatment with venetoclax and 5-azacitidine in direct comparison.

Patient ID	Age	Gender	WHO category	Karyotype	Cytogenetic risk score (Schanz *et al.*)	Severe anemia	IPSS category	IPSS-r category	*ASXL1* mutation	*RUNX1* mutation	*TP53* mutation	*EZH2* mutation	Previous therapy	IWG Response (Cheson *et al.*)
1	84	male	sAML	46, XY	Good	absent	sAML	sAML	no mut	n.d.	no mut	n.d.	no treatment	Naive
2	66	male	RAEB-1	46, XY	Good	present	intermediate 1	high	mut	n.d.	n.d.	n.d.	EPO	Naive
3	74	male	RCMD	46, XY	Good	present	intermediate 1	intermediate	no mut	no mut	no mut	n.d.	no treatment	Naive
4	75	male	RCMD (from RAEB-2)	46, XY	Good	Present	complete remission	complete remission	mut	no mut	no mut	no mut	HMA	Marrow CR
5	68	male	RCMD (from sAML)	46, XY	Good	Present	intermediate 1	low	n.d.	n.d.	n.d.	n.d.	HMA	Marrow CR
6	85	male	sAML	46, XY	Good	absent	sAML	sAML	no mut	no mut	no mut	no mut	HMA	Stable disease
7	76	female	sAML	46, XX	Good	absent	sAML	sAML	n.d.	n.d.	n.d.	n.d.	HMA	Stable disease
8	76	male	sAML	46, XY	Good	present	sAML	sAML	no mut	no mut	no mut	no mut	HMA	Stable disease
9	71	male	sAML	46, XY,+1,der(1;22)(q10;q10)	Intermediate	absent	sAML	sAML	mut	no mut	no mut	no mut	HMA	Failure
10	80	male	RAEB-1	46, XY	Good	absent	intermediate 1	high	n.d.	n.d.	n.d.	n.d.	HMA	Failure
11	70	male	sAML	46, XY	Good	present	sAML	very high	no mut	no mut	no mut	no mut	HMA	Failure
12	58	female	sAML	46,XX,del(5q),del(16q)	Good	absent	sAML	sAML	n.d.	n.d.	n.d.	n.d.	HMA	Failure
13	57	male	RAEB-2	46, XY	Good	absent	intermediate 2	high	no mut	no mut	no mut	no mut	HMA	Failure
14	71	male	RAEB 1	46, XY; 45, XY, -7	Bad	absent	intermediate 2	very high	n.d.	n.d.	n.d.	n.d.	HMA	Failure
15	75	male	RAEB 2	46, XY	Good	present	intermediate 1	high	mut	mut	n.d.	n.d.	HMA	Failure
16	66	female	sAML	46,XX,del(5)(q22q3?3),der(18)t(14;18)(q2?;q2?)[9]//46,XY[1]	Good	present	sAML	sAML	n.d.	n.d.	n.d.	n.d.	HMA	Failure
17	80	male	EB-2	47, XY del5q, r6, +8, del13q, der16; 46, XY	Very bad	present	intermediate 2	very high	no mut	no mut	mut	no mut	HMA	Failure
18	68	male	EB-1	46, XY	Good	present	intermediate 1	high	mut	mut	n.d.	n.d.	HMA	Failure
19	68	male	EB-1	46, XY	Good	present	intermediate 1	high	mut	mut	n.d.	n.d.	HMA	Failure
20	74	female	sAML	complex (>3 aberrations)	Very bad	absent	sAML	sAML	no mut	no mut	mut	no mut	HMA	Failure
21	73	female	RAEB-2	complex (>3 aberrations)	Very bad	absent	intermediate 2	very high	n.d.	n.d.	mut	n.d.	HMA	Failure

Table S1:

Clinical and molecular characteristics of patients with Myelodysplastic Syndromes (MDS) and secondary Acute Myeloid Leukemia (sAML) contributing samples. Age, gender, WHO category, karyotype, cytogenetic risk score (according to Schanz *et al.*), presence or absence of severe anemia (defined as hemoglobin <8g/dl in females and <9g/dl in males), IPSS category, IPSS-*r* category and the presence or absence of mutations in *ASXL1, RUNX1, TP53* or *EZH2,* as well as previous treatment and response (according to IWG response criteria (Cheson *et al.*)) were documented for each patient sample. Patients with sAML were defined by >20% bone marrow blast infiltration. Mutational status of MDS and sAML samples was determined by conventional Sanger sequencing or next-generation sequencing. Abbreviations: MDS with multilineage dysplasia (MDS-MLD), MDS with blast excess <10% (EB-1), MDS with blast excess > 10% (EB-2), not done (n. d.), hypomethylating agent (HMA).

The mean age of the MDS/sAML cohort is 72.14 (range 57–84 years) and of the HMA failure cohort is 70.08 (range 57–80 years) compared to 64.96 (range: 49-85 years) of the healthy control. The p value comparing age MDS/sAML HMA failure vs. age healthy control is 0.0859. Comparing the age of healthy controls to the age of all MDS/sAML samples analyzed the p value is 0.0067.
