# Supplementary material for: Venetoclax with azacitidine targets refractory MDS but spares healthy hematopoiesis at tailored dose
Source: Exp Hematol Oncol. 2019 Apr 16;8:9. doi: 10.1186/s40164-019-0133-1 (PMC6469098; doi:10.1186/s40164-019-0133-1)
Supplement: Supplementary file 2 — Additional file 2. Patients and Methods. [file 40164_2019_133_MOESM2_ESM.pdf]

Patients and Methods

Patient samples

Human bone marrow (BM) samples were collected according to the institutional guidelines and in concordance with the Declaration of Helsinki. Written informed consent was obtained from each patient. The investigation was approved by the Local Ethics Committee of the University Hospital of the Technical University in Munich (Vote #62/16S). sAML was defined as $>$20% of blasts in the BM and a history of MDS. Samples were obtained when clinically required from patients either before or during treatment and irrespective of the therapeutic regimen. Control samples were obtained from human femoral heads discarded after implantation of total endoprosthesis of the hip joint from hematologically healthy age-matched donors.

Cell isolation and culture

Mononuclear cells from primary human BM samples were isolated via density-gradient centrifugation using the Biocoll Separation Solution (Biochrom AG, Berlin, Germany) following the manufacturer's instructions. $CD34^+$ cells were purified via positive selection using the $CD34^+$ MicroBeads kit (Miltenyi Biotec, Bergisch Gladbach, Germany) and purity was confirmed to be at least 95%. BM mononuclear cells (BMMNCs) were cultured at a density of 5×10^5 cells/ml in serum-free media consisting of Iscove's Modified Dulbecco's Medium (IMDM) with L-alanyl-L-glutamine (IMDM GlutaMAX) with 20% BIT 9500 serum substitute (1% (w/v) bovine serum albumin, 10µg/ml insulin, 200µg/ml iron-saturated transferrin; StemCell Technologies, Vancouver, BC, Canada) and enriched with recombinant human stem cell factor (100ng/ml), FMS-related tyrosine kinase-3 ligand (100 ng/ml), thrombopoietin (10 ng/ml), interleukin-6 (5 ng/ml), interleukin-3 (10 ng/ml; all from R&D Systems, Minneapolis, MN, USA), β-mercaptoethanol (10 µM; Gibco, Carlsbad, CA, USA) and low-density lipoproteins (4 µg/ml; Sigma-Aldrich, St Louis, MO, USA).

Inhibitors

5-azacitadine (5-AZA) was kindly provided by the central pharmacy of TUM's university hospital and Venetoclax (ABT-199) was kindly provided by AbbVie Inc. (North Chicago, Illinois, U.S.A.). Both inhibitors were dissolved in dimethyl sulfoxide (DMSO) and used in final concentrations from 1µM to 10µM as specifically noted in the figures and texts. DMSO was used at 0.001% as vehicle control.

Colony formation assay

Hematopoietic progenitors were assessed after treatment with 5-AZA and/or Venetoclax in final concentrations from 1µM to 10µM or DMSO (0.001%) for 72 h in cytokine-supplemented, serum-free culture. 1×10^4 BMMNCs were plated in duplicates in methylcellulose medium supplemented with an optimal cytokine mix according to the manufacturer's protocols (MethoCult H4435 enriched; StemCell Technologies). Numbers of erythroid progenitor colonies (Burst-forming units-erythroid or colony-forming units for the granulocytic-macrophagic lineage, and multi-potential granulocytic-erythroid-macrophagic-

megakaryocytic lineage) were assessed after 14 days. Transmitted light photographs were obtained on a Keyence BIOREVO BZ-900 microscope.

Flow cytometry

BMMNCs were stained with Annexin V-FITC in Annexin V staining solution (0.1M HEPES/NaOH, pH 7.4, 1.4M NaCl 0.9%, 25mM $CaCl_2$), followed by staining with fluorescently labeled antibodies against CD34 (clone 4H11) and CD45 (clone 2D1). Dead cells were excluded by 7-aminoactinomycin D (7AAD) staining. If not otherwise stated, reagents and antibodies were purchased from eBioscience. Flow analysis was performed on a BD FACS Canto II (BD Bioscience) and data were analyzed using FlowJo software (TreeStar Inc., Ashland, OR, USA).

Statistical analysis

Each patient received several treatments. Differences between two treatments were assessed using the paired t-test with significance level of 5%. For Figure 2a the unpaired t-test was used when analyzing the whole cohort. Statistical analyses were performed using GraphPad Prism version 6.0g (Graphpad Software, Inc., San Diego, CA, USA).
